# High-resolution melting curve analysis for rapid detection of mutations in a Medaka TILLING library

**DOI:** 10.1186/1471-2199-11-70

**Published:** 2010-09-15

**Authors:** Tomoko Ishikawa, Yasuhiro Kamei, Shinji Otozai, Jinhyong Kim, Ayuko Sato, Yoshikazu Kuwahara, Minoru Tanaka, Tomonori Deguchi, Hidenori Inohara, Tohru Tsujimura, Takeshi Todo

**Affiliations:** 1 Department of Radiation Biology and Medical Genetics, Graduate School of Medicine, Osaka University, B4, 2-2 Yamadaoka, Suita, Osaka 565-0871, Japan; 2 Department of Otorhinolaryngology and Head and Neck Surgery, Osaka University School of Medicine, Osaka 565-0871, Japan; 3 Department of Pathology, Hyogo College of Medicine, Nishinomiya 663-8501, Japan; 4 Department of Pathology, Institute of Development, Aging and Cancer, Tohoku University, Seiryo-machi 4-1, Aoba-ku, Sendai 980-8575, Japan; 5 Laboratory of Molecular Genetics for Reproduction, National Institute for Basic Biology, Okazaki 444-8787, Japan; 6 Research Institute for Cell Engineering (RICE), National Institute of Advanced Industrial Science and Technology (AIST), 3-11-46 Nakouji, Amagasaki, Hyogo 661-0974, Japan

## Abstract

**Background:**

During the last two decades, DNA sequencing has led to the identification of numerous genes in key species; however, in most cases, their functions are still unknown. In this situation, reverse genetics is the most suitable method to assign function to a gene. TILLING (Targeting Induced Local Lesions IN Genomes) is a reverse-genetic strategy that combines random chemical mutagenesis with high-throughput discovery of the induced mutations in target genes. The method has been applied to a variety of plant and animal species. Screening of the induced mutations is the most important step in TILLING. Currently, direct sequencing or nuclease-mediated screening of heteroduplexes is widely used for detection of mutations in TILLING. Both methods are useful, but the costs are substantial and turnaround times are relatively long. Thus, there is a need for an alternative method that is of higher throughput and more cost effective.

**Results:**

In this study, we developed a high resolution melting (HRM) assay and evaluated its effectiveness for screening ENU-induced mutations in a medaka TILLING library. We had previously screened mutations in the *p53 *gene by direct sequencing. Therefore, we first tested the efficiency of the HRM assay by screening mutations in *p53*, which indicated that the HRM assay is as useful as direct sequencing. Next, we screened mutations in the *atr *and *atm *genes with the HRM assay. Nonsense mutations were identified in each gene, and the phenotypes of these nonsense mutants confirmed their loss-of-function nature.

**Conclusions:**

These results demonstrate that the HRM assay is useful for screening mutations in TILLING. Furthermore, the phenotype of the obtained mutants indicates that medaka is an excellent animal model for investigating genome stability and gene function, especially when combined with TILLING.

## Background

Our understanding of the basic mechanisms underlying most biological processes has been transformed by the systematic application of mutational analysis. Traditionally, forward genetics, driven by the identification of mutant phenotypes, has been the most widely used approach. On the other hand, genome sequencing projects over the past few decades have identified numerous genes in key species, and the completion of these sequences produced a situation in which most of the genes are known, but most of their phenotypes are obscure. In this situation, reverse genetics, which provides targeted inactivation of genes identified by sequence analysis followed by phenotype analysis of the mutant, has become an important tool for many biologists. In mice, reverse genetics is usually carried out using homologous recombination in embryonic stem cells, which allow a precise mutation to be constructed in nearly any gene. However, embryonic stem cells are only available in a limited number of organisms. Thus, a general method that is applicable to many organisms would be in great demand, and several approaches have been tried.

One of these approaches is TILLING (Targeting Induced Local Lesions IN Genomes). TILLING is a reverse-genetic strategy that combines random chemical mutagenesis with high-throughput discovery of the induced mutations in target genes. The method is general and, following its original application to the model plant *Arabidopsis thaliana *[1,2], has been applied to a variety of plant and animal species including maize, lotus, barley, wheat, *Drosophila*, zebrafish, and medaka [3-9]. The first step in TILLING is chemical mutagenesis. For mutagenesis in animals, males are mutagenized using N-ethyl-N-nitrosourea (ENU) and then used to generate a large population of F1 animals that consequently harbor many random heterozygous mutations in their genomes. Next, the DNA from these animals is analyzed for mutations in a specific gene of interest. Once a mutation is identified, homozygous mutant animals can be obtained by crossing progeny from heterozygous F1 matings.

Among the vertebrates, small laboratory fish are suitable for the study of gene function due to their ease of handling, large numbers of progeny per generation, and, in particular, their translucent embryos. In many species, embryos develop outside the mother's body, enabling easy visual inspection and manipulation of their tissues and cells. One such fish is the zebrafish, *Danio rerio*, which is the most widely used laboratory fish. The success of forward genetics in the past two decades has established the zebrafish as the premier vertebrate for the study of gene function. Medaka, *Oryzias latipes*, is another small laboratory fish that has been used as an experimental model animal since the 1920s. Medaka has a small genome size (one-half of that of zebrafish) and is phylogenetically distinct from zebrafish, having diverged about 110 million years ago [10], making it useful for comparisons of conserved and divergent gene function in teleost evolution. For these reasons, both the zebrafish and medaka are currently widely used in comparative mutagenesis, carcinogenesis, and genomic instability studies. Shimada and Shima [11] showed that the mutation response of the medaka male germ cell is comparable to that of the mouse, and therefore medaka could serve as a vertebrate model system of DNA damage response in germ cells.

All cells have elaborate mechanisms to protect their genomes. DNA can be damaged by reactive metabolic byproducts and by environmental mutagens, and the response to DNA damage and its repair are crucial for cell viability and disease prevention. The DNA-damage response is a signal-transduction pathway that coordinates cell-cycle transitions, DNA replication, DNA repair, and apoptosis [12,13]. The major regulators of the DNA-damage response are two protein kinases, ataxia-telangiectasia mutated (ATM) and ATM and Rad3-related (ATR) [14-17]. These two large kinases have significant sequence homology and play important roles in the initiation of cell cycle checkpoints by targeting an overlapping set of substrates that promote cell-cycle arrest and DNA repair [18]. Another key player that mediates cell cycle checkpoints is the tumor suppressor p53, which is activated and stabilized by ATM and serves as a transcription factor for the downstream cell cycle regulator [19]. The p53 pathway depends on the transcription of its downstream genes and its activity is therefore delayed after DNA damage. Thus, its role lies in the maintenance of the cell cycle checkpoint.

The most widely used reverse genetics' tool in zebrafish and medaka is undoubtedly morpholinos, but they are not used as a substitute for mutations because they can only be used in transient methods and for early developmental stages. Recently, TILLING was applied to zebrafish, and several mutants have been described [8]. The success of this reverse genetics approach in zebrafish allowed us to apply TILLING to medaka. Once males have been mutagenizaed with ENU, the TILLING procedure can be divided into two steps, construction of a library and screening of the library. The library consists of pair sets of genomic DNA and frozen sperm of F1 offspring from each ENU-mutagenized male. We established a medaka TILLING library from 5771 F1 male fish derived from ENU-mutagenized G0 [9,20].

In the next screening step, two methods are widely used for the detection of mutations, direct sequencing [9] and *Cel*I nuclease-mediated screening for heteroduplex formation with wild-type and mutant alleles [2]. Both methods are effective; however, costs are substantial and turnaround time is relatively long. These disadvantages have resulted in the development of alternative methods that are more cost effective, faster, and easier to perform. The high resolution melting (HRM) analysis is a recently developed, promising technology used for the detection of variations in DNA [21,22]. It is an in-tube method that can be performed in a fast, cheap, and robust manner. The thermal stability of a DNA fragment is determined by its base sequence. When the DNA fragment contains an altered sequence, the duplex stability is changed, leading to different melting behavior, which can be identified with HRM analysis. During HRM analysis, melting curves are produced using intercalating DNA dyes that fluoresce in the presence of double-stranded DNA and a specialized instrument designed to monitor fluorescence during heating [23,24]. When the temperature increases, the DNA-intercalating dye is released from the DNA and the fluorescence decreases. This process produces a characteristic melting profile that can be monitored with precision. Changes in the sequence within the DNA fragment, as in SNPs or mutations, alter the melting profile. The HRM assay has successfully been used to detect point mutations in ethyl methanesulfonate-mutated plant populations [25].

In the present study, we developed HRM assays to evaluate the effectiveness of this methodology for screening ENU-induced mutations in the TILLING library. We first used the HRM assay to screen mutations in exons 5 and 6 of the *p53 *gene because the same library was previously screened with the direct sequencing method [9], enabling comparison of the two methods. We detected mutations in exons 5 and 6 of *p53 *with efficiency equivalent to that of the direct sequencing method. Then we screened for mutations in the *atr *and *atm *genes. We identified 15 mutations in *atr *and 11 in *atm*, including one nonsense mutation in each gene. The phenotypes of these nonsense mutants confirmed their loss-of-function nature. The results demonstrate the utility of the HRM assay for mutant screening using TILLING.

## Results

### Screening of mutations in p53 gene

**Figure 1:**
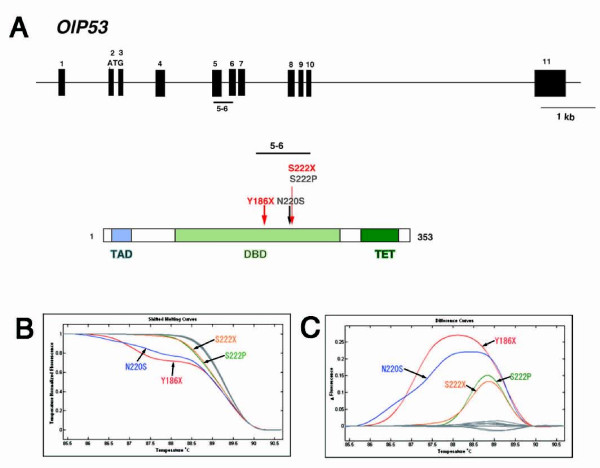
**ENU-induced mutations identified by TILLING in the *Oryzias latipes p53 *gene (OlP53)**. (A) Genome organization and protein structure of the medaka *p53 *gene. The region analyzed by PCR and the HRM assay is indicated by the horizontal bar in the top panel; the protein is shown below. Numbers in the top panel refer to exons; numbers in the bottom panel refer to amino acids. The ENU-induced mutations are shown by arrows. TAD: transactivation domain, DBD: DNA-binding domain, TET: tetramerization domain. High-resolution melting curves (B) and subtraction plots (C) of identified ENU-induced mutations in exons 5 and 6 of the p53 gene in the TILLING genomic DNA.

To evaluate whether the HRM assay is applicable to the screening of a TILLING library, we first used it to screen in exons 5 and 6 of the *p53 *gene (Fig. 1) and compared the results with those obtained previously by screening the same library with direct sequencing [9]. The *p53 *HRM assay yielded 5 HRM-positive samples that displayed aberrant melting curves. To magnify the changes in the contour of the melting curve, subtraction plots are generated from the melting curve by subtracting the test curve from that of the wild type (see Materials and Methods). Examples of the melting curves and subtraction plots are shown in Figs. 1B and 1C, respectively. Subsequent sequencing of the positive samples revealed mutations (Table 1) that were correctly identified by the HRM assay except for three mutations, N220D, E241X, and a splicing site mutation (Table 2). Of these, E241X and the splicing site mutation were located close to the end of the primer used for PCR amplification (Additional file 1, Fig. S1). A previous report demonstrated that the sensitivity (error rate) of HRM scanning does not depend on the position of the mutation within the PCR product when the mutations are positioned more than 50 bp from each end [26], but our result shows a lower sensitivity for mutations located near the end of the PCR products. Interestingly, the HRM assay identified two additional mutations, an intronic mutation and S222X, which were not identified by the previous sequencing method. The sensitivity of the HRM assay was 62% (5/8), which is considerably lower than the 75% (6/8) for the sequencing. However, when the PCR primers were designed far enough from each end of the target exon, the sensitivity improved to 83% (5/6), which is adequate for screening the TILLING library.

**Table 1:** Mutations identified in the present study.

Gene	Exons Screened	Amplicons	Base-pairs screened	Exonic			Intronic		Total	Mutation rate (Base-pairs/One mutation)
							
				Stop	Missense	Silent	Intron	Splice		
p53	2	1	1702800	2	2	0	1	0	5	340560
ATM	5	4	7740932	1	14	3	9	0	27	286701
ATR	7	4	7837612	1	10	6	6	0	23	340766

total	14	9	17281344	4	26	9	16	0	55	314206

**Table 2:** p53 mutations detected by sequencing and HRM.

Sequence context	Type of mutation or amino acid change*	
	
	SEQ-positive	HRM-positive
5'-TGGCCCAGTA(T > A)TTTGAAGACC-3'	Y186X	Y186X
5'-CCGCAGGTAA(A > T)GCTGCCCCAG-3'		Intron
5'-CTACATGTGT(A > G)ACAGCTCGTG-3'	N220D	
5'-TACATGTGTA(A > G)CAGCTCGTGC-3'	N220S	N220S
5'-GTGTAACAGC(T > C)CGTGCATGGG-3'	S222P	S222P
5'-GTGTAACAGCT(C > A)GTGCATGGG-3'		S222X
5'-TCTGGAAACC(G > T)AGTAAGTTTA-3'	E241X	
5'-GGAAACCGAG(T > C)AAGTTTAGTC-3'	Splice	

*X indicates a stop codon.

### Screening of mutations in atr and atm genes

**Figure 2:**
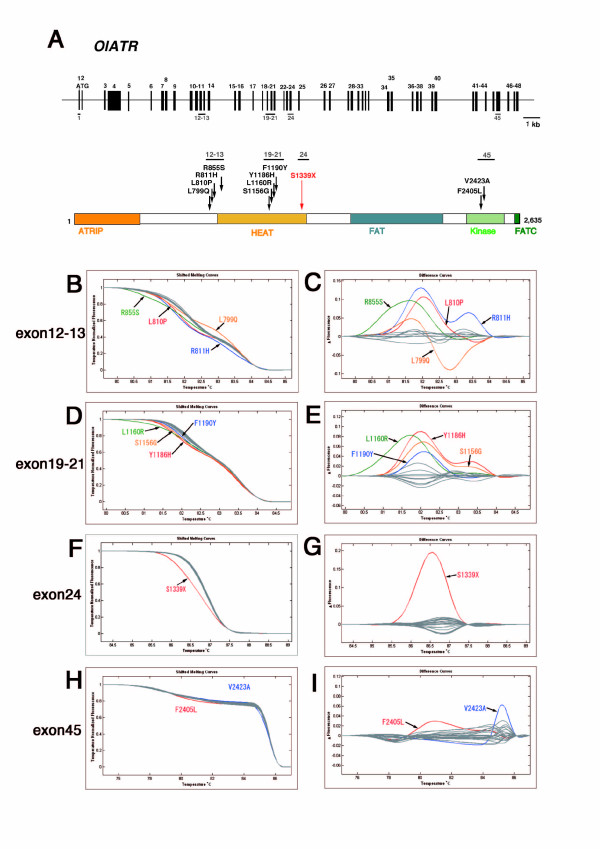
**ENU-induced mutations identified by TILLING in the *Oryzias latipes atr *gene (OlATR)**. (A) Genome organization and protein structure of the medaka *atr *gene. Conventions are as in Fig. 1. The ATRIP binding domain, HEAT domain, FAT domain, PI3-kinase domain (kinase), and FATC domain are shown in the protein. High-resolution melting curves (B, D, F, H) and subtraction plots (C, E, G, I) of identified ENU-induced mutations in exons 12-13, 19-21, 24, and 45 of the *atr *gene are shown.

**Figure 3:**
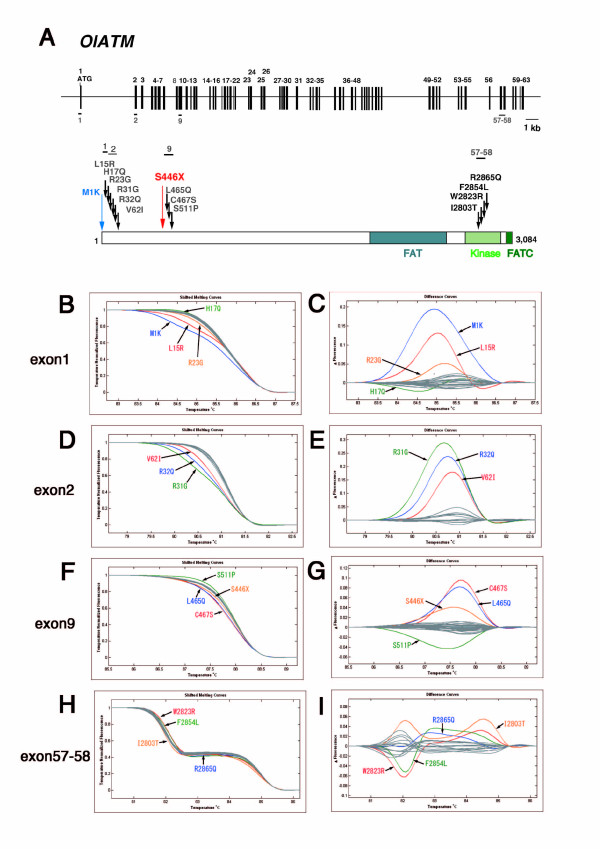
**ENU-induced mutations identified by TILLING in the *Oryzias latipes atm *gene (OlATM)**. (A) Genome organization and protein structure of the medaka *atm *gene. Conventions are as in Fig. 1. The FAT domain, PI3-kinase domain (kinase), and FATC domain are shown. High-resolution melting curves (B, D, F, H) and subtraction plots (C, E, G, I) of identified ENU-induced mutations in exons 1, 2, 9, and 57-58 of the *atm *gene are shown.

**Figure 4:**
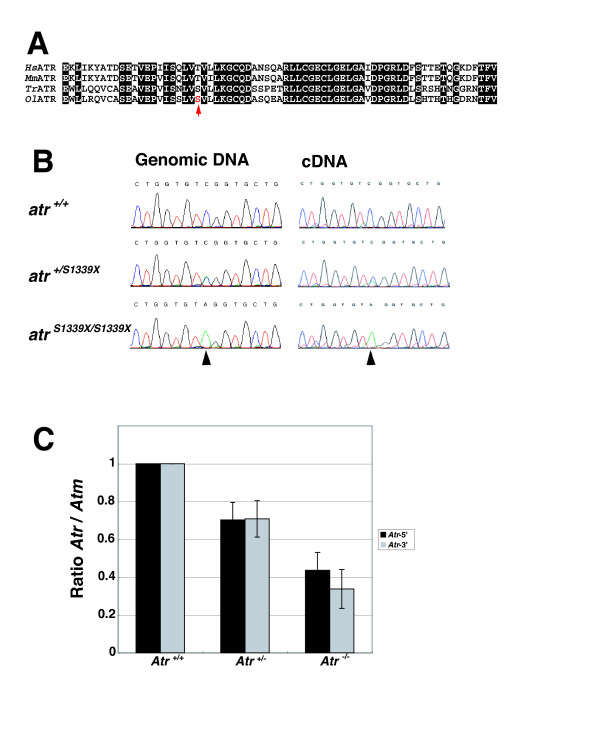
**Molecular characterization of the medaka *atrS1339X *mutant**. (A) Sequence alignment of vertebrate ATR (*Hs, Homo sapiens; Mm, Mus musculus; Tr, Takifugu rubripes; Ol, Oryzias latipes*) in exon 24. Conserved residues are highlighted by white letters on a black background. Red letter and arrow indicate the mutation site. (B) Sequence traces of the region of the the point mutation (arrows) in the founder fish. (C) Quantitative PCR showed a decrease to 70% of wild type in transcripts of heterozygous *atr *mutants and a decrease to 40% in homozygous mutants. Ratios of *atr *cDNA to *atm *cDNA as a control are shown, each normalized to the levels in the respective wild-types. Quantitative PCR was performed either at the 5' region (black bars) or 3' region (gray bars).

We next performed the HRM assay to screen for mutations in the *atr *and *atm *genes for each of 4 different amplicons covering 6 and 5 exons, respectively (Figs. 2, 3 and Additional file 2, Table S1). After subsequent sequencing of HRM-positive PCR amplicons, we identified 23 independent mutations in *atr *and 27 in *atm *(Tables 1 and 3). Figs. 2 and 3 show melting curves and subtraction plots from the HRM assay of PCR amplicons with amino-acid-substitution-type mutations. We retrieved highly likely loss-of-function mutations for each gene by screening for nonsense mutations, and found point mutations in exon 24 of *atr *and in exon 9 of *atm *that created premature stop codons in the proteins (S1339X and S444X for *atr *and *atm*, respectively). These two exons were well conserved when compared with the corresponding exons of mammals and teleost *atr *or *atm *genes (see Figs. 4A and 8A). To demonstrate their loss-of-function nature, heterozygotes obtained by artificial insemination were backcrossed twice to wild-type fish, and then homozygous mutants for each mutation were established by mutual crossing of the heterozygotes.

**Table 3:** Summary of ATM and ATR mutations detected by HRM.

Gene	Exon	Sequence context	Amino acid change
ATM	1	5'-TGAAGCAGGA(T > A)GAGTCTGGCT-3'	M1K
	1	5'-TGCAGAGGGC(T > G)GGAACATGAT-3'	L15R
	1	5'-GGCTGGAACA(T > A)GATAAAGCTA-3'	H17Q
	1	5'-AGCTACTGAG(A > G)GAAAGGTAAA-3'	R23G
	2	5'-TGAAAACTTC(A > G)GACGGCTCCT-3'	R31G
	2	5'-AACTTCAGAC(G > A)GCTCCTTCGA-3'	R32Q
	2	5'-ATGGGACAAT(G > A)TCTTCAGGTA-3'	V62I
	9	5'-CCACTGCTGT(C > A)GGCGTTGCAC-3'	S444X
	9	5'-CTGTACGTGC(T > A)GCGCTGCCTC-3'	L463Q
	9	5'-CGTGCTGCGC(T > A)GCCTCAGGGA-3'	C465S
	9	5'-CCACACAGAG(T > C)CTCTCAGCCT-3'	S509P
	57	5'-ACCATGCCCA(T > C)CGGGGAGTTT-3'	I2792T
	57	5'-ACCTCAGGAT(T > A)GGACCAGCCT-3'	W2812R
	58	5'-TTGCAAAAAC(T > C)TCAGACCGGT-3'	F2843L
	58	5'-TGCATGGAAC(G > A)ATTCCTAGAC-3'	R2854Q

ATR	12	5'-CATGTGAACC(T > A)GACCAGGGAA-3'	L799Q
	12	5'-CAGGCGGTTC(T > C)CCGTTCTCTG-3'	L810P
	12	5'-GCGGTTCTCC(G > A)TTCTCTGACC-3'	R811H
	13	5'-CCTGGTCGCG(C > A)GTCTGAAGGA-3'	R855S
	20	5'-GGCTTTGACG(A > G)GTGTGATGGC-3'	S1156G
	20	5'-GTGATGGCTC(T > G)GATGCGCCTG-3'	L1160R
	20	5'-CGGCCTCCGA(T > C)ACAAAGAAGA-3'	Y1186H
	20	5'-AAAGAAGACT(T > A)CCCTCTGCTG-3'	F1190Y
	24	5'-AGCCTGGTGT(C > A)GGTGCTGCTG-3'	S1339X
	45	5'-CGGCTCCCTT(T > A)GAGGAGAAAC-3'	F2405L
	45	5'-CATCCGCCGG(T > C)CTTCCATGAG-3'	V2423A

### atr is required for cellular proliferation

**Figure 5:**
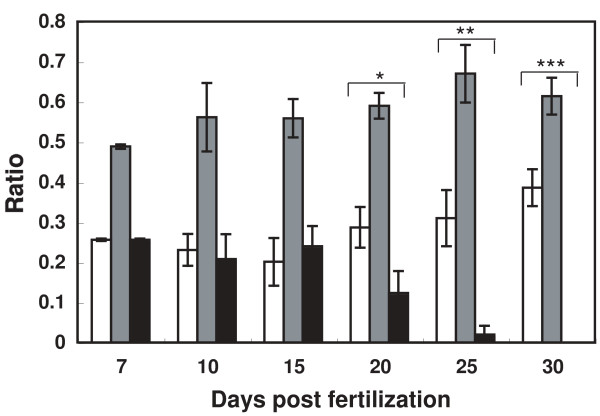
**Characterization of the medaka *atrS1339X *mutant**. Genotypic ratios of embryos and fry from heterozygous intercrosses. *atr*^*+/S1339X *^fishes were intercrossed and eggs were collected. After culture for the indicated number of days, the genotypes of surviving eggs (80-100) were determined. Data are means ± SD. Differences in the ratios of each genotype from mendelian inheritance ratios at each day were tested with the χ^2 ^test. Day 20, *P *< 0.05; day 25, *P *< 10^-5^; day 30, *P *< 10^-7^. Black bars: *atr *^*S1339X/S1339X*^, gray bars: *atr*^*+/S1339X*^, white bars: *atr*^*+/+*^.

In mice, *Atr *is essential for early embryonic development, and *Atr*^-/- ^embryos die in early developmental stages [27,28]. To test whether *atr *is also essential for development in medaka, we generated homozygous *atr*-deficient fishes by intercrossing *atr*^*+/S1339X *^fishes and determined their viability. Surprisingly, *atr*^*S1339X/S1339X *^embryos developed and hatched successfully. No apparent abnormality was observed under the stereomicroscope in the *atr*^*S1339X/S1339X *^embryo during development. The hatched fry survived at rates similar to the wild-type and *atr*^*+/S1339X *^fishes for 15 days after fertilization; however, they subsequently started to die gradually, and none of the *atr*^*S1339X/S1339X *^mutants was as alive at day 30 after fertilization (Fig. 5). The growth of the hatched *atr*^*S1339X/S1339X *^fry was slightly poorer than that of the wild type (average body height of *atr*^*S1339X/S1339X *^fry = 0.48 mm at day 15, which is shorter than 0.58 mm of *atr*^*+/+*^or *atr*^+/S1339X^; Additional file 3, Fig. S2); this was associated with a high rate of generalized edema, followed by death on days 20-25. These results indicate that *atrS1339X *mutation produces milder phenotype in medaka than in mice. However, we also have to consider possible involvement of undetected ENU-induced mutations in the phenotype observed for *atr*^*S1339X/S1339X *^mutant. We cannot exclude possibility that undetected ENU-induced mutations still remain after several backcrossing to wild type fish and the remaining mutation affect the phenotype.

These viable fry were used to verify the knockout phenotype. We could not confirm the knockout phenotype at the protein level in the *atr*^*S1339X/S1339X*^, due to the lack of an appropriate anti-ATR antibody that recognizes the medaka protein. However, we were able to show the knockout phenotype by quantitative PCR. Quantitative PCR was carried out using two sets of PCR primers; each was designed to amplify the 5' or 3' region of the *atr *transcript. Both primer sets yielded the same results. The *atr *transcript levels decreased to ~70% of the wild-type for heterozygous mutants and ~40% for homozygous mutants, presumably due to nonsense-mediated decay of the transcript containing the premature stop codon (Fig. 4C). Finally, we confirmed the nonsense mutation in the transcript by sequencing the cDNA from the homozygous mutant (Fig. 4B).

### The atmS444X mutant is sensitive to radiation, but partially infertile

**Figure 6:**
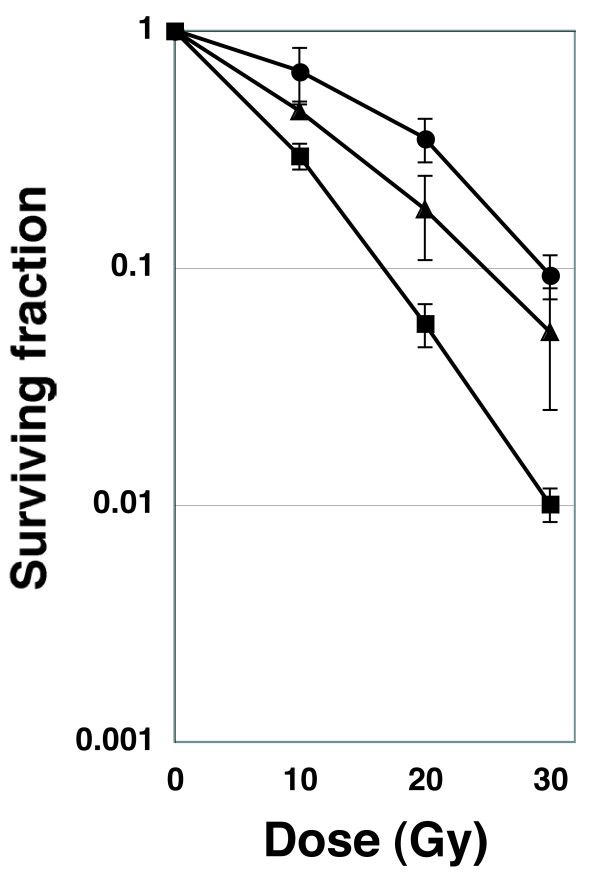
**Clonogenic survival of medaka cells exposed to 10, 20, or 30 Gy of γ-rays**. Colonies were counted and plotted (mean ± SEM). Circles: cells established from *atm*^*+/+*^, triangles: cells established from *atm*^*+/S444X*^, squares: cells established from *atm *^*S444X/S444X*^.

Patients with the recessive disease ataxia telangiectasia and *atm*-deficient mice are sensitive to ionizing radiation [15,29-31]. To determine the radiosensitivity of the *atm*^*S444X/S444X *^mutant, we established culture cell lines from *atm*^*+/+*^, *atm*^*+/S444X*^, or *atm*^*S444X/S444X *^embryos and determined their radiosensitivities by their ability to form colonies. As expected, *atm*^*S444X/S444X *^cells displayed increased sensitivity to X-rays, as manifested by a profound fall in survival rate with increased exposure dose (Fig. 6).

**Figure 7:**
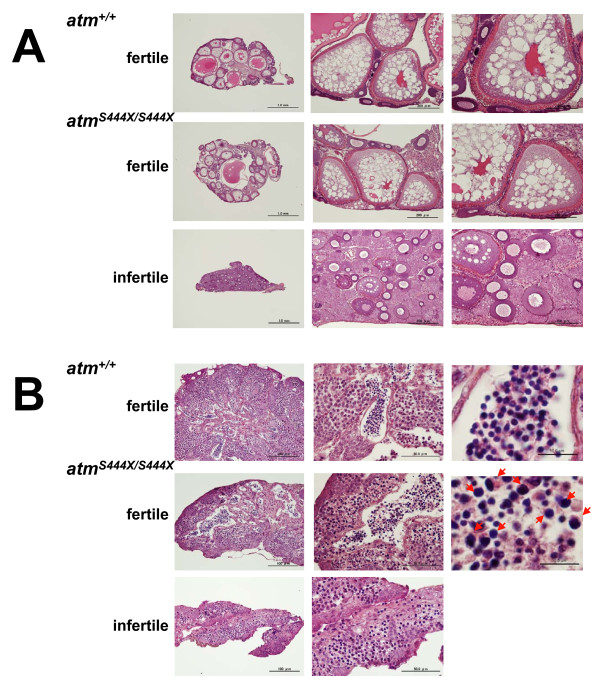
**Defective spermatogenesis and oogenesis in *atm***^***S444X/S444X***^**fish**. (A) Histological sections of ovaries of 16-18 week-old *atm*^*+/+ *^and *atm *^*S444X/S444X *^littermates were stained with hematoxylin-eosin. Scale bars; left panels, 1.0 mm; middle panels, 200 μm; right panels, 100 μm. (B) Hematoxylin-eosin stained sections of the seminiferous region of 16- to 18-week-old *atm*^*+/+ *^and *atm *^*S444X/S444X *^fishes. Spermatids and/or sperm showing abnormal size are indicated by arrowheads in the rightmost middle panel. Scale bars; left panels, 1000 μm; middle panels, 50 μm; right panels, 10 μm.

Another apparent phenotype of *Atm*-deficient mice is infertility due to severe disruption of gametogenesis in both males and females [29-31]. To determine the fertility of fish homozygous for the *atm *disruption, we mated mutant and wild-type fish. However, surprisingly, both male and female *atm*^*S444X/S444X *^fish were only partially infertile. When mated with wild-type males, 25% of *atm*^*S444X/S444X *^females spawned eggs. The number of spawned eggs was normal and almost all the fertilized eggs developed normally. In contrast, when mated with wild-type females, 57% of *atm*^*S444X/S444X *^males were able to fertilize the eggs, but the fertilization rate was extremely low compared with the wild type. On average, the fertilization rate of *atm*^*S444X/S444X *^males was 1.16% versus 93.4% in the wild-type male. Histological examination of ovaries from wild-type adult females showed oocytes at various phases of development (Fig. 7A, top panels). In contrast, ovaries of infertile mutants contained no mature oocytes and disorganized interstitial cells (Fig. 7A, bottom panels), whereas those from the fertile mutant fish showed normally developed oocytes, similar to the wild type (Fig. 7A, middle panels). Interestingly, ovaries of the infertile mutant seemed to have hyperplastic germ cell-clusters among the disorganized interstitial cells. The cell-clusters resembled cysts formed by several rounds of pre-meiotic divisions in normal development [32]. Differences in testicular structure were also observed between the wild type and mutants (Fig. 7B). The normal testicular architecture of the wild type showed the presence of cells in all stages of spermatogenesis (e.g., spermatogonia, spermatocytes, spermatids, and mature sperm in the lobules (Fig. 7B, top panels)). The testes of the infertile mutants contained spermatogonia and spermatocytes, but not spermatids or sperm (Fig. 7B, bottom panels). The testes of fertile mutant fish contained cells in all stages of spermatogenesis, but the spermatids and sperm were abnormal in size; in the testes of the wild-type fish all spermatids and sperm were quite uniform in size, but they varied in size in the testes of the mutant fish (more than 50% of the cells were larger than those in wild-type male, Fig. 7B, right panels). These results suggest the presence of a unique damage response mechanism in medaka. However, as in the case of the *atr*^*S1339X/S1339X *^mutant, we also have to consider possible involvement of undetected ENU-induced mutations in the phenotype observed for *atm*^*S444X/S444X *^mutant. We cannot exclude possibility that undetected ENU-induced mutations still remain after several backcrossing to wild type fish and the remaining mutation affect the phenotype.

**Figure 8:**
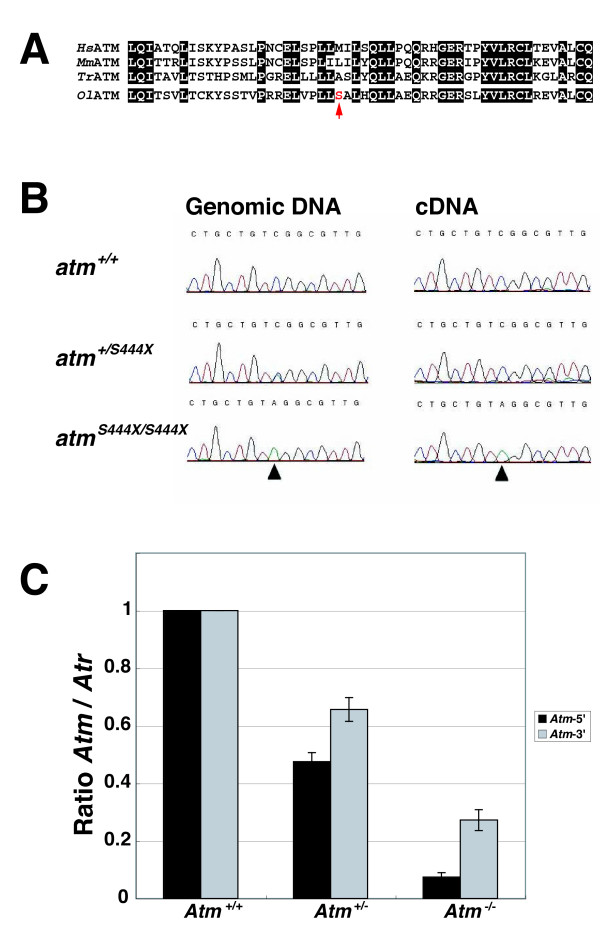
**Molecular characterization of the medaka *atmS444X *mutant**. (A) Sequence alignment of vertebrate ATM in exon 9. Conserved residues are highlighted by white letters on a black background. (B) Sequence traces of the region of the point mutation in cultured cells established from the wild type and heterozygous and homozygous *atmS444X *mutants. (C) Quantitative PCR showed a decrease in the *atm *transcript in *atmS444X *heterozygous and homozygous cells. Ratios of *atm *cDNA to *atr *cDNA as a control are shown, normalized to the levels in the respective wild-type proteins. Quantitative PCR was performed either at the 5' region (black bars) or 3' region (gray bars).

**Figure 9:**
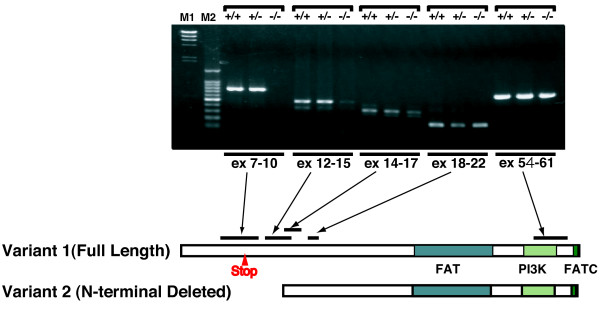
**RT-PCR amplification of the *atm *cDNA at different exons**. The cDNAs were synthesized from wild-type (*atm*^*+/+*^), *atm*^*+/S444X *^(*atm*^*+/-*^), or *atm*^*S444X/S444X *^cells (*atm*^*-/-*^) and used as substrates for the PCR amplification of each exon. Left two lanes are DNA size markers, M1: *Hind*III-digested λ DNA; M2: 100-bp ladder.

We next examined the knockout phenotype of *atm*^*S444X/S444X *^by quantitative PCR. Interestingly, the 5' and 3' PCR primer set yielded different quantitative values. The level of *atm *transcript determined by quantitative PCR using the 3' primer set was ~66% of the wild-type in heterozygous mutants and ~27% in homozygous mutants (Fig. 8C, gray bars). The decrease was even more pronounced when the 5' PCR primer was used: ~50% of wild type in heterozygous mutants and ~7% in homozygous mutants (Fig. 8C black bars). Furthermore, the S444X mutant allele was not detected in the cDNA obtained from *atm*^*+/S444X *^cells, as confirmed by sequencing, although both S444X and S444 wild alleles were detected in the genomic DNA (Fig. 8B*atm*^*+/S444X*^). These results suggest that transcripts containing the S444X mutation remain at very low levels in *atm*^*S444X/S444X *^cells due to extensive decay of the transcript containing the premature stop codon, but *atm *transcripts containing the 3' region still remain at substantial levels in the same cells. In fact, amplification of the cDNA fragment containing the S446X mutation (exon 9) of *atm*^*S444X/S444X *^cells was very difficult. Only after three repeated nested PCRs we were able to obtain sufficient PCR product for sequencing (Fig. 8B*atm*^*S444X/S444X*^). One possible explanation for the difference between the 5' and 3' quantitative PCR is the presence of a short transcript that lacks the N-terminal coding region involving the S444 coding site but contains the 3' *atm *coding region. To test this possibility and to determine which region of the *atm *transcript is present in *atm*^*S444X/S444X *^cells, we amplified several exons in the *atm *gene by RT-PCR using cDNA from the wild-type, heterozygous, and homozygous mutant cells. We successfully amplified cDNA fragments containing exons 14-17, exons 19-20, and exons 55-56 in cDNA from all type of cells, whereas cDNA fragments containing exons 7-10 and exons 12-15 could not be amplified in cDNA from *atm*^*S444X/S444X *^cells, suggesting the presence of a short transcript that starts from the region around exon 12-13 (Fig. 9). Consistent with this notion, we found a full-length-cDNA clone (DK103188) starting from exon 13 by searching a full-length-cDNA database (http://www.shigen.nig.ac.jp/medaka/). The starting sequence in the cDNA was also confirmed by 5' RACE (data not shown). Taken together, these results indicate the existence of two *atm *transcript variants in medaka cells. These variants encode a longer isoform 1 and a shorter isoform 2 that has a truncated N-terminus compared to isoform 1.

## Discussion

The critical step in the TILLING procedure is the screening for mutations, but to successfully identify mutations in the target genes, it is necessary to have an efficient mutation discovery system. The TILLING library is constructed from F1s derived from chemical mutagen-treated G0s, and thus the mutations induced in each F1 are heterozygous; therefore, methods that quickly distinguish heteroduplex DNA from the homoduplex counterpart can be applied to the discovery of these mutations. HRM was recently introduced as a screening method for mutation detection and reported to be useful for heteroduplex detection [21,22]. It is an in-tube method that can be performed in a fast, cheap, and robust manner. To evaluate the HRM assay as an alternative mutation screening method for TILLING, in this report we used the HRM assay to screen a total of 17.3 Mb in a medaka TILLING library. We identified 55 independent mutations (Table 1) and found that the average mutation frequency of the library was 1 mutation per 314 kb. The value is slightly higher than that obtained by direct sequencing the same library; 1 mutation per 345 kb [9]. The higher rate of mutation detection by the HRM assay relative to that by the direct sequencing method is unexpected because direct sequencing is the gold standard for identifying mutations and thus would be expected to detect mutations more efficiently than any other method. Misclassification of SNPs by the HRM assay might be the reason for the discrepancy. The genomic DNA library used contains a substantial level of SNPs. When the same base change was identified more than twice, it was classified not as a mutation but as a SNP (see Materials and Methods). In the HRM assay, screening was carried out using 96-well PCR plates. Melting curve analysis was performed on each plate, but comparison between plates could not be performed. If one type of SNP appeared only once in one plate and appeared more than twice in other plates, the former was misclassified as a mutation and confirmed by sequencing, whereas the latter were correctly classified as SNPs and not subjected to checking by sequencing. However, misclassification could not have been common because the mutation detection rate was only slightly higher in the HRM assay than in the direct sequencing method. Despite this issue of classification, our results demonstrate the utility of the HRM assay for the screening of mutations in the TILLING library.

In this report, we used the HRM assay to identify medaka mutants producing truncated forms of the ATM or ATR proteins, *atmS444X *and *atrS1339X*, respectively. *Atm *and *Atr *orchestrate overlapping DNA damage responses to different forms of DNA strand discontinuities. However, knockout mouse models have also suggested that *Atr*, in contrast to *Atm*, is essential for viability, and that *Atr*-null mice die at an early embryonic stage [27,28]. Interestingly, *atr*^*S1339X/S1339X *^medaka can continue normal development during embryogenesis and hatch successfully, but the hatched fry all die before day 30 postfertilization. We tried to establish a culture cell line from *atr*^*S1339X/S1339X *^embryos, but the cells died within a few weeks and no cell line was obtained (data not shown), a finding similar to results with cells from *Atr*-null mice [27,28]. It is not clear at present why *atr*-deficient embryos can survive for several weeks; however, the viable *atr *fry provide a good model system for analyzing *atr *function at the organismal level.

Meiosis is a specialized cell division program to generate hemizygous gametes. An important feature of meiosis is the exchange of genetic information between maternal and paternal chromatids, a process called meiotic recombination [33,34]. Meiotic recombination is initiated by Spo11-catalyzed meiotic DNA double-strand breaks (DSBs), followed by their repair using an intact homologous non-sister chromatid [35]. DSBs are highly hazardous for genome integrity, but meiotic cells deliberately introduce them into their genome in order to initiate homologous recombination. Therefore, DSBs left unrepaired are checked by a stringent surveillance mechanism, the meiotic recombination checkpoint, which delays meiosis I until DSB repair is completed. Another important genome surveillance mechanism is the DNA damage checkpoint, which senses DSBs that arise at unpredictable locations as a consequence of DNA damage during both mitosis and meiosis [13,33,36,37]. Mechanistically, the meiotic recombination checkpoint is related to the DNA damage checkpoint; however, many aspects of the meiotic recombination checkpoint are considerably less well understood than those of mitotic DNA damage checkpoint. Given the well established role of ATM in DSB repair in mitosis, ATM might play an important role in the signaling and processing of the programmed meiotic DSBs. In this study, we found defective meiosis in the medaka *atm*^*S444X *^mutant. The striking difference between *Atm *mice and the mutant fish is the fertility of the latter; the *Atm *mice are completely infertile, whereas the mutant fish are partially infertile, and 25% of females and 57% of males remain fertile. In mice, *Atm *deficiency results in early arrest in prophase I followed by apoptotic degeneration, which results in the complete absence of mature gametes and spermatocytes in various stages of degeneration [29,30,38]. Similar premeiotic arrest was observed in tissue sections of the infertile mutant fish, but the spermatocytes did not show any stage of degeneration (Fig. 7B). Instead, a high percentage of abnormally large spermatids and/or sperm were noted in the testes of the fertile mutants. These observations suggest that, in medaka, many cells bearing unrepaired meiotic DSBs are removed by apoptosis, but a substantial fraction proceed to completion of meiosis, resulting in the production of abnormal sperm. In fact, a similar partially sterile phenotype has been reported in *Arabidopsis ATM *mutants [39], whose *ATM *meiocytes did not appear to undergo apoptosis before the end of meiosis despite the observed chromosomal fragmentation during anaphase I and II. The histology presented in the current report suggests that the infertility in *atm*-deficient medaka is possibly due to disorganized gonads in both males and females. At present, the precise role of the meiotic recombination checkpoint defect in *atm *medaka in causing the reproductive defects is not clear. More precise characterization of the abnormal sperm and oocytes should provide important information about the meiotic recombination checkpoint in *atm *mutant fish.

Finally, we should emphasize again the possible involvement of undetected ENU-induced mutations in the observed phenotype. The single major drawback of TILLING as a method for reverse genetics is that a mutation of interest that is identified in any given F1 fish is only one of many heterozygous mutations in its genome. By multiplying the estimated size of medaka genome by the mutation rate detected in the present TILLING genomic DNA library, an estimate of the number of ENU-induced mutations per F1 fish is ~2300. More than 80% of these mutations were removed after backcrossing to wild-type fish three times, however significant levels of mutations still remain. As a result, we cannot exclude possibility that the remaining mutations affect the phenotype. Most strong evidence for the direct relationship between the observed phenotype and the mutated gene is the phenotype rescue with the wild-type gene. Further studies including the phenotype rescue with BAC (Bacterial artificial chromosome) clone, will clarify a possible involvement of these unknown mutations.

ATM contains three domains that are important for its function, a FAT domain, a protein kinase domain, and a FATC domain, which are positioned in the C-terminal half of the protein. In addition to these domains, several known ATM substrates bind to the substrate binding site, a region near the N-terminus of the protein [15]. The N-terminus is a crucial region of the ATM because its deletion inactivates the protein [40,41]. In this study, we demonstrated the presence of a short *atm *transcript variant in medaka cells that encodes an isoform (isoform 2) lacking the N-terminal 767 residues of the full-length ATM protein. A similar short isoform has also been reported in human cells (GenBank Accession No. NM 138242 or Ensemble Transcript: ENST00000452508). These short isoforms lack the substrate binding site and thus cannot complement the radiosensitivity of *atm*-deficient mutants. Mutations in ataxia telangiectasia patients are well characterized: over 200 different *ATM *mutations have been described, and approximately 85% of the mutants reported in ataxia telangiectasia patients are predicted to truncate the protein, but the mutations are mapped uniformly over the gene, which includes the N-terminal region truncated in the short isoform [41,42]. Therefore, at present we do not know the function of the short isoform, and there is no answer to why the short isoform is conserved in both human and fish. However, the medaka *atm*^*S444X/S444X *^mutant provides an excellent model for analyzing the function of this short *atm *isoform.

## Conclusions

In the present study we demonstrate that the HRM assay is useful for screening mutations in TILLING. The present study also demonstrates that mutations in medaka *atr *affect viability and in *atm *affect fertility and radiosensitivity, similar to findings in mice and humans. However, the phenotypes of these mutants show milder effects in medaka than in mammals. On the basis of our results and those of others, we conclude that medaka is a useful model for the analysis of genome stability, and can be used to complement studies in zebrafish, mouse and human.

## Methods

### Medaka TILLING Library

Mutagenesis was carried out as described previously [9,43]. Briefly, 102 male *Kyoto-Cab*, a substrain of Cab, were mutagenized by 3 consecutive treatments every week with 3 mM ENU (Sigma-Aldrich, St. Louis, MO). One month after the last ENU treatment, 97 surviving fertile fish were crossed with wild-type females to obtain the mutagenized F1 male library. The number of offspring produced from a single mutagenized male founder varied from 1 to 239. The sperm from each F1 medaka was cryopreserved, as described previously (Medaka Book, http://www.shigen.nig.ac.jp/medaka/medakabook/). After removal of the testes, the fish was kept at -80°C until DNA was extracted. The posterior half of the fish was incubated overnight at 55°C in lysis buffer containing 10 mM Tris-HCl (pH 7.5), 1 mM EDTA (pH 8.0), 120 mM sodium chloride, 12 mM sodium citrate (pH 7.0), 1% SDS, and 200 μg/mL proteinase K. The lysate was extracted with phenol and precipitated with isopropanol. The DNA pellet was dissolved in 1 mL TE (10 mM Tris pH 7.5, 1 mM EDTA, pH 8.0). The concentration was adjusted to 4 ng/μL, aliquoted into 96-deep-well plates, and stored at -20°C. Finally, sets of frozen sperm and genomic DNA were prepared from 5771 F1 males and used as the TILLING library.

### Identification of mutations by HRM analysis

#### Primers

BLAST searches of medaka databases (http://www.ensembl.org/Oryzias_latipes/index.html) using the human *ATR *or *ATM *gene revealed one homologous gene each (*Olatr *or *Olatm*, respectively) in the medaka genome and no paralogous genes. The degrees of conservation with the human *ATR *or *ATM *are 59.4% or 51.9% identity, respectively. Each genomic DNA in the library was amplified by PCR with gene-specific primers. To design the gene-specific primers, parts of the cDNA sequences of *atm *and *atr *were determined by RT-PCR and used to retrieve the genomic sequences from two Medaka databases (http://www.ensembl.org/Oryzias_latipes/index.html and http://medaka.utgenome.org/). The following 3 criteria were used for selection of the target exon for each gene: 1) location in the N-terminal region, 2) correspondence to the region encoding conserved amino acid residues, and 3) sufficient length (more than 150 base pairs). The first two criteria were employed to obtain null-function mutants. The third criterion was selected for high cost performance. We designed the primer set using Light Scanner Primer Design software (Idaho Technology, Salt Lake City, UT, USA). The designed primers were further checked for simulated melting curves using MeltSim 1.0-beta for Windows (http://bioinformatics.org/meltsim/wiki/). The primers used are summarized in Additional file 2, Table S1.

#### PCR

PCR reactions were performed in 96-well microtiter plates in 7-μL volumes. Reactions included 7 ng of genomic DNA in 1 × KOD Plus PCR buffer (Toyobo, Tokyo), with 1 mM MgSO4, 200 μM of deoxynucleotide triphosphate, 0.6 × LC Green PLUS (Idaho Technology), 0.14 U of KOD Plus polymerase (Toyobo, Tokyo), and 300 nM of each primer. DMSO (10% vol/vol) was added when the amplified region was GC-rich. Reactions were overlaid with 10 μL of mineral oil (Nacalai, Kyoto, Japan), the plates were centrifuged (1500 × *g *for 3 min), and PCR was performed in an iCycler (Bio-Rad Laboratories, Hercules, CA) or PXE0.2 Thermal Cycler (Thermo Scientific, Rockford, IL). The cycling and melting conditions were set as follows: one cycle of 94°C for 2 minutes, followed by 45 cycles of 94°C for 15 seconds; a primer-specific annealing temperature for 25 seconds, and then 68°C for 25 seconds; and a final denaturing and re-annealing step (one cycle of 94°C for 30 seconds followed by rapid cooling to 28°C. The final step was included to redistribute DNA strands derived from mutant and wild-type alleles in order to maximize heteroduplex formation.

#### HRM analysis

After PCR, the plates were centrifuged (1500 × *g *for 3 min) and imaged in a 96-well LightScanner (Idaho Technology). The plates were heated at 0.3°C/second, and fluorescence measurements were collected from 65°C to 98°C. Melting curves were analyzed as described previously [44] using the LightScanner software. Briefly, after exponential background subtraction, melting curves were normalized between 0% and 100%. Normalized and temperature-overlaid curves were viewed on the subtraction plots to magnify differences in the shapes of the melting curves. The subtraction plots were generated by subtracting each curve from the mean wild-type curve, defined as the most common genotype. The subtraction plot helps to cluster the samples into groups. Clustering of the melting curves for genotype identification was performed manually using the LightScanner software on high sensitivity.

The fluorescence data generated during DNA melting can be analyzed based on the melting temperature (Tm) or on the shape of the melting curve. Most homozygous sequence changes produce a Tm shift compared to the wild type [45]. In contrast, heterozygous samples are identified not by product Tm, but by differences in melting curve configuration [46]. In the TILLING library, all the induced mutations were heterozygous. PCR amplification of heterozygotes followed by heat denaturing and annealing resulted in the formation of 4 duplexes: two homoduplexes and two heteroduplexes. Each duplex has a characteristic melting temperature, and the sum of all transitions can be observed by melting curve analysis. The subtraction plot was used to detect changes in the contour of the melting curve.

HRM screening of the TILLING library was carried out in two steps. First, each of the 5771 genomic DNAs was assayed by HRM, and the genomic DNAs showing melting curves different from the wild-type allele were selected as mutant candidates (first positive). The HRM assay was repeated for the first positive genomic DNAs, and the genomic DNAs showing melting curves different from the wild-type allele were picked up again as second positives. Proof of induced mutation was obtained by sequencing the PCR product of the second positive genomic DNAs. As reported previously [9], substantial numbers of SNPs exist in the genomic DNA of the library used. Therefore, the second positive DNAs included both SNPs and true mutations. SNPs were discriminated from true mutations by defining the same type of base change detected in more than two sequencing reactions as an SNP.

### Sequencing

PCR products containing the second positive HRM amplicons were purified using a Sephadex G50 (Fine DNA Grade) column and then used as a template for the sequencing reaction. Sequencing reactions were carried out by using BigDye Terminator version 3.1 (Applied Biosystems, Foster City, CA) and the ABI 3730xl sequencing platform.

### Generation of atm and atr mutant fish

F2 fish carrying the *atmS444X *or *atrS1339X *mutation were obtained by artificial insemination using the frozen sperm from the TILLING library. Artificial insemination was performed as described previously [47]. Briefly, about 100 unfertilized eggs were obtained from the wild-type females. A single glass capillary containing 10 μL of sperm from the F1 fish identified in the screening was removed from the liquid nitrogen and thawed at ambient temperature. Immediately after thawing, the content was placed in balanced salt solution (BSS; 0.65% sodium chloride, 0.04% potassium chloride, 0.02% magnesium sulfate heptahydrate, 0.02% calcium chloride dihydrate, 0.00005% phenol red, 0.01% sodium hydrogen carbonate, pH 7.3) and incubated with eggs for 20 minutes with occasional pipetting. Eggs that were not fertilized were removed 3 h later, and BSS was replaced with 0.03% Red Sea salt water. The eggs were incubated at 28°C until hatching. Artificial insemination was conducted using *atmS444X *or *atrS1339X *sperm with a Cab or HdrR female, respectively. Each resultant fish was crossed out twice to wild-type Cab or HdrR fish, respectively. Three independent in-crosses of heterozygous progeny were then performed, from which homozygous mutant males were obtained and used for this study.

Fish were genotyped by PCR analysis or sequencing of fin-clip DNA. For PCR genotyping, PCR reactions were performed using two primer sets, wild-type and mutant, which have the wild-type or mutant allele sequence, respectively, at the 3' end. The primers used for genotyping are listed in Additional file 4, Table S2. For genotyping by sequencing, PCR reactions were performed with the same primers as used for the screening of the library.

### Primary cell culture

Cell cultures were established from embryos as described previously (http://www.shigen.nig.ac.jp/medaka/medakabook/). Cells were cultured at 27°C. Briefly the embryos were sterilized for at least 40 s in Dakin's solution (58 mM NaOCl, 6 mM HCl, and 93 mM NaHCO_3_), washed in phosphate-buffered saline (PBS), resterilized for 10 s in 70% ethanol, and washed in PBS. The chorion and yolk sac were removed from each embryo, transferred to L-15 medium, and minced using a 22G needle connected to a 1-mL syringe to obtain fibroblast-like cells. Cells were cultured in L-15 medium supplemented with 10% fetal bovine serum (Gibco BRL, Grand Island, NY), 50 mg/mL streptomycin, 50 U/mL penicillin, and 10 mM 2-[4-(-hydroxyethyl)-1-piperazinyl] ethanesulfonic acid (HEPES; pH 7.5), and incubated at 27°C.

### Sensitivity to X-ray irradiation

Cultured cells were exposed to γ-rays from a ^137^Cs source at a dose rate of 0.93 Gy/min (Gamma Cell 6000 Elan, MDS Nordion, Ottawa, Canada). OLCAB-e3 cells were irradiated with γ-rays at the dose of 100 Gy and used for feeder cells. After irradiation, *atm*^*+/+ *^or *atm*^*S444X/S444X *^cells were cultured in 6-well plates in the presence of feeder cells (7 × 10^5 ^cells/well) and incubated at 27°C. Two weeks after irradiation, the cells were fixed with ethanol and stained with 0.006% crystal violet, the formed colonies were counted, and the survival rate was calculated.

### Survival of ATR^-/- ^fish

*atr*^*+/S1339X *^fishes were intercrossed and eggs were cultured at 27°C in a 10-cm plastic dish. After culture for 7, 10, 15, 20, 25, or 30 days, the genotypes of surviving eggs or fry (80-100 for each fraction) were determined by PCR or by sequencing.

### Histopathological analysis

The gonads were dissected and fixed in Bouin's solution. Fixed tissues were embedded in paraffin blocks. Cross-sections were cut at a 5-μm thickness and stained with hematoxylin-eosin. Sections were examined and photographed under light microscopy.

### Reverse-transcription PCR and quantitative PCR

RNA was isolated from cultured cells established from mutant or wild-type fish using Sepasol RNAI Super (Nacalai Tesque) and reverse transcribed by using the ReverTra Ace^R ^qPCR kit (Toyobo). For *atr *and *atm *quantitative PCR, cDNA-specific primers were designed to amplify either the 5' or 3' region of the *atm *and *atr *transcripts (5' regions were within exons 1-3, 3-4, 7-8 for *atr *and exons 2-3 for *atm*; 3' regions were within exons 47-48 for *atr *and exons 33-34 for *atm*). The sequences of all oligonucleotides that were used are listed in Additional file 4, Table S2.

## Contribution of Authors

TI and YKa participated in the design and performance of all experiments described in this paper. In addition, TI wrote the first draft of the first manuscript. SO, JK, and HI participated in mutant screening with the HRM assay. AS and TTs performed histology, and TD participated in identification of the *atr *phenotype. YKu and MT provided expertise on histological examinations of fish gonads and supervised their interpretation. HI and TTo secured funding for the experiments. TTo conceived the project; designed the experimental plan; and supervised the design, execution, and interpretation of all experiments.

## Supplementary Material

Additional file 1 **Figure S1: Location of PCR primers (horizontal arrows) used for screening of mutations in *p53 *exons 5 and 6.** Mutations are indicated with vertical arrows. Click here for file

Additional file 2 Table S1: Primer sequences for screening of mutations. Click here for file

Additional file 3 **Figure S2: Growth of *atr*^*+/+*^*, atr*^*+/S1339X*^, or *atr*^*S1339X/S1339X *^fishes.** (A) Images of representative embryos and fry of each genotype are shown. Body length (B) and body height (C) of 92 fry were measured and plotted. Click here for file

Additional file 4 Table S2: Primer sequences for genotyping, real-time PCR, and RT-PCR. Click here for file
