# Supplementary figures and images for: High-resolution melting curve analysis for rapid detection of mutations in a Medaka TILLING library
Source: BMC Mol Biol. 2010 Sep 15;11:70. doi: 10.1186/1471-2199-11-70 (PMC2949603; doi:10.1186/1471-2199-11-70)

**A**

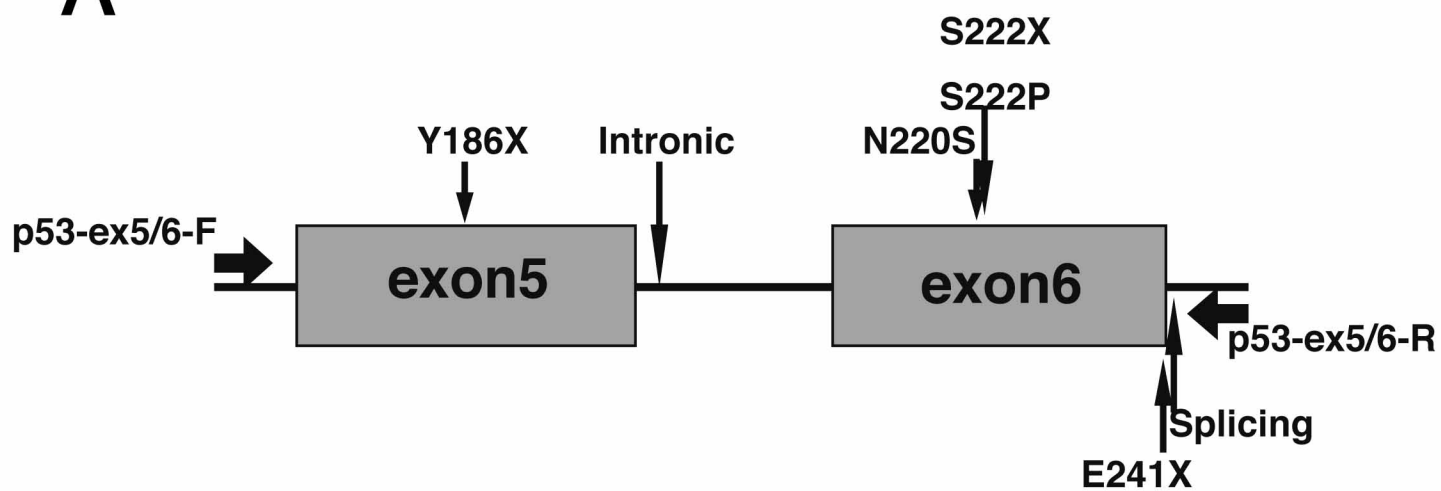

**B**

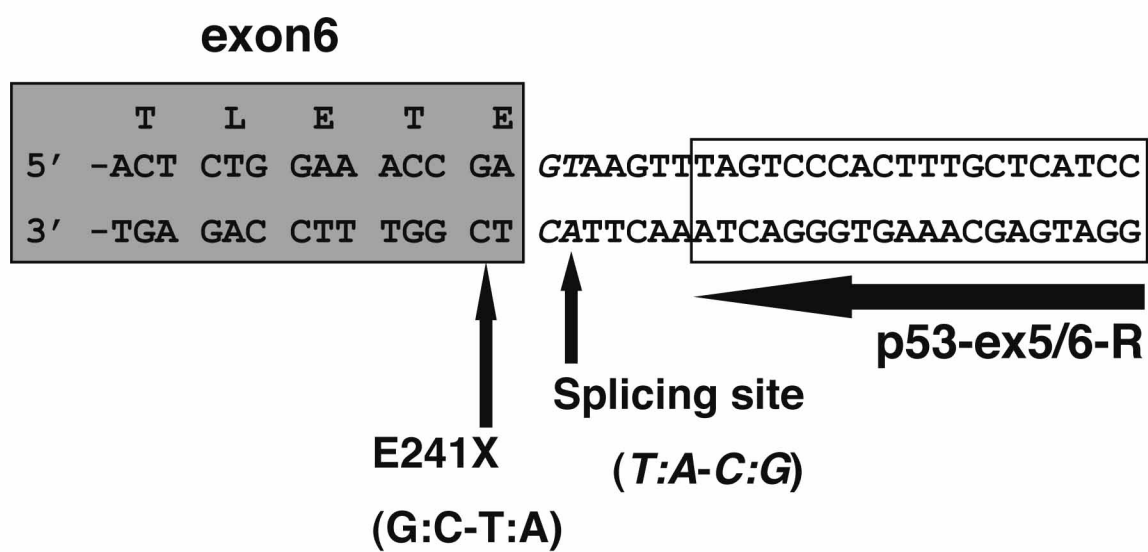

Supplement: Additional file 1 — Figure S1: Location of PCR primers (horizontal arrows) used for screening of mutations in p53 exons 5 and 6. Mutations are indicated with vertical arrows. [file 1471-2199-11-70-S1.PDF]
