# Supplementary material for: High-resolution melting curve analysis for rapid detection of mutations in a Medaka TILLING library
Source: BMC Mol Biol. 2010 Sep 15;11:70. doi: 10.1186/1471-2199-11-70 (PMC2949603; doi:10.1186/1471-2199-11-70)
Supplement: Additional file 2 — Table S1: Primer sequences for screening of mutations. [file 1471-2199-11-70-S2.PDF]

Additional file 2, Table S1. Primer sequences for screening of mutations.

Gene	Exon	Primer name	Sequence	Amplicon size (bp)
p53	5, 6	p53-ex5/6-F	TTTCTCCATCGACTGTTACATTT	343
		p53-ex5/6-R	GGATGAGCAAAGTGGGACTA	
ATM	1	ATM-ex1-F	TGCATTTTGCCTTGAATGGA	287
		ATM-ex1-R	GGGATAGGCTCCAGGGATTC	
	2	ATM-ex2-F	AGAACACCTTGTGGGGCATT	348
		ATM-ex2-R	TTCTTCACCATGCGGTTGTC	
	9	ATM-ex9-F	CAAGCATGTATTCTCTGTCTTTAGG	419
		ATM-ex9-R	GCACATTAAAACAAATAACTCACTTG	
	57, 58	ATM-ex57/58-F	GACGTTGTGGTTCACACTCG	487
		ATM-ex57/58-R	TGCAGGATGTATTGGGTCAA	
ATR	12, 13	ATR-ex12/13-F	GCTACAAGTGGCGTCAAGCA	540
		ATR-ex12/13-R	GGTGCTGAGACCGAGGAAGA	
	19, 21	ATR-ex19/21-F	TGAATCCTTGCTCAGTTGGTCTT	676
		ATR-ex19/21-R	TTCAGGGATCAGTTTCATTTAGCA	
	24	ATR-ex24-F	CTGGTGCGGTAAGCACTGC	264
		ATR-ex24-R	CGGTTTAAAGAGTGGGGTATCAC	
	45	ATR-ex45-F	AACCGAGTCGTTGATTTCTCTC	400
		ATR-ex45-R	GGAGTGGGTCTTTAAAACTTAAATATG	
