# Supplementary material for: High-resolution melting curve analysis for rapid detection of mutations in a Medaka TILLING library
Source: BMC Mol Biol. 2010 Sep 15;11:70. doi: 10.1186/1471-2199-11-70 (PMC2949603; doi:10.1186/1471-2199-11-70)
Supplement: Additional file 4 — Table S2: Primer sequences for genotyping, real-time PCR, and RT-PCR. [file 1471-2199-11-70-S4.DOC]

Additional Table S2. Primer sequences for genotyping, real-time PCR, and RT-PCR.

| For Genotyping | |  |  |
| --- | --- | --- | --- |
| Gene | Mutant | Primer name | Sequence |
| ATM | S446X | ATM-ex9-F | CAAGCATGTATTCTCTGTCTTTAGG |
|  |  | ATM-S446X-WT-R | AGCTGGTGCAACGCC***G*** |
|  |  | ATM-S446X-Mut-R | AGCTGGTGCAACGCC***T*** |
| ATR | S1339X | ATR-ex24-F | CTGGTGCGGTAAGCACTGC |
|  |  | ATR-SX-WTR | AGCCCTTCAGCAGCACC***G*** |
|  |  | ATR-ex24-R | CGGTTTAAAGAGTGGGGTATCAC |
|  |  | ATR-SX-Mut-F | GGTCATCTCCAGCCTGGTGT***A*** |
| For Real-time PCR | |  |  |
| Gene | Exon | Primer name | Sequence |
| ATM | 1-3 | ATM-5'-1F | TGCAGAGGGCTGGAACATGATA |
|  | 1-3 | ATM-5'-1R | CCTGGTCCTGAAGACATTGTCC |
|  | 3-4 | ATM-5'-2F | GCAGAGGGAAGTACGCCACA |
|  | 3-4 | ATM-5'-2R | CGTATGGCTCCCACTGTCATC |
|  | 7-8 | ATM-5'-3F | CAGAAGAAGATGGCTGAAGTGTG |
|  | 7-8 | ATM-5'-3R | TTCAGCAGCTCACTGCAATTC |
|  | 47-48 | ATM-3'-2F | TGGCTGAAACCTGTCTGGAA |
|  | 47-48 | ATM-3'-2R | GAAGCGAGCCAGAGACAAGAA |
| ATR | 2-3 | ATR-5'-1F | ATCCTGACCGATGTGGATGT |
|  | 2-3 | ATR-5'-1R | GTATTCAGCAGGGTGGCAAG |
|  | 33-34 | ATR-3'-1F | CCTGGAAGCTCGGAAAGTG |
|  | 33-34 | ATR-3'-1R | GTCTCAGCGTCCTGCTTTTG |
| For RT-PCR | |  |  |
| Gene | Exon | Primer name | Sequence |
| ATM | 7-10 | ATM-ex5L | GCTCTGTTCGGCTTTTTCAC |
|  |  | ATM-ex11R | AAGCCAACTCTTGTGTCTTTCA |
|  |  | ATM-ex7L | AGCTCACGGCTGACATCTG |
|  |  | ATM-ex10R | CTCCCAAAGAGTCCCAGATG |
|  | 12-15 | ATM-ex12L | AGCAGCTTGTGCTTTCTGTG |
|  |  | ATM-ex15R | GATGAGGCTGGCTGGTAGAG |
|  | 14-17 | ATM-ex14L | AAGGCGCCATGTCTAGTCTC |
|  |  | ATM-ex17R | CCTCTGCCAGCAGACATTTT |
|  | 19-20 | ATM-ex18L | ACCTTCCTGCTGAAGACTCG |
|  |  | ATM-ex22R | GGCATCATCTCCCTCATTTT |
|  |  | ATM-ex19L | TCTGTGCGGTCTGTATCGTC |
|  |  | ATM-ex20R | GCTCTCACAGACGCAGAACA |
|  | 55-60 | ATM-ex54L | GGATGGCAAGAGCAGGAGACA |
|  |  | ATM-ex61R | CCAATCAAACAGAGGGTCGT |
|  |  | ATM-ex55L | CTTCAGCATGTGCTCCATGT |
|  |  | ATM-ex60R | TCCACAATGGTCAACAGAGC |
